# Supplementary material for: Synthesis of piperidines and pyridine from furfural over a surface single-atom alloy Ru1CoNP catalyst
Source: Nat Commun. 2023 Oct 10;14:6329. doi: 10.1038/s41467-023-42043-6 (PMC10564752; doi:10.1038/s41467-023-42043-6)
Supplement: Supplementary file 1 — Supplementary Information [file 41467_2023_42043_MOESM1_ESM.pdf]

Synthesis of Piperidines and Pyridine from Furfural over a Surface Single-Atom Alloy Ru_1Co_{NP} Catalyst

Haifeng Qi,[1,2#] Yurou Li,[3#] Zhitong Zhou,[1] Yueqiang Cao,[3] Fei Liu,[1] Weixiang Guan,[1] Leilei Zhang,[1] Xiaoyan Liu,[1] Lin Li,[1] Yang Su,[1] Kathrin Junge,[2] Xuezhi Duan,[3,*] Matthias Beller,[2,*] Aiqin Wang,[1,4,*] Tao Zhang[1,4]

[1] CAS Key Laboratory of Science and Technology on Applied Catalysis, Dalian Institute of Chemical Physics, Chinese Academy of Sciences, Dalian, 116023, China.

[2] Leibniz-Institut für Katalyse, Albert-Einstein-Straße 29a, Rostock 18059, Germany.

[3] State Key Laboratory of Chemical Engineering, East China University of Science and Technology, Shanghai, 200237, China.

[4] State Key Laboratory of Catalysis, Dalian Institute of Chemical Physics, Chinese Academy of Sciences, Dalian, 116023, China.

[#] These authors contributed equally to this work.
* Corresponding authors: X. Duan (xzduan@ecust.edu.cn)
M. Beller (matthias.beller@catalysis.de)
A. Wang (aqwang@dicp.ac.cn)

Inventory of Supporting Information

Supplementary Methods....3

Supplementary Figure 1....6

Supplementary Figure 2....7

Supplementary Figure 3....8

Supplementary Figure 4....9

Supplementary Figure 5....10

Supplementary Figure 6....11

Supplementary Figure 7....12

Supplementary Figure 8....13

Supplementary Figure 9....14

Supplementary Figure 10....15

Supplementary Figure 11....16

Supplementary Figure 12....17

Supplementary Figure 13....18

Supplementary Figure 14....19

Supplementary Table 1....21

Supplementary Table 2....22

Supplementary Table 3....23

Supplementary Table 4....24

Supplementary Table 5....25

Supplementary Table 6....26

Supplementary Table 7....27

Supplementary Table 8....28

Supplementary References....29

Supplementary Methods

Catalyst preparation. All catalysts were prepared by incipient wetness impregnation. As an example, for the preparation of Ru_1Co_{20}/HAP catalyst, 209 mg $Co(OAc)_2·4H_2O$ and 135 mg 3.17 wt_{Ru}% $RuCl_3$ were added to 1.4 g water and sonicated for 10 min, followed by addition of 1 g HAP (Calcined at 500 °C for 2 h). Then the mixture was evaporated under freeze drying for 12 h. The obtained solid was ground to powder and then transferred to the tube furnace and then heated to 400 °C in air atmosphere at a ramp of 5 °C/min and was held at that temperature for 2 h. After cooling to room temperature, the gas was switched to hydrogen atmosphere and the tube was fluxed with hydrogen for 30 min. The tube furnace was then heated to 400 °C in hydrogen atmosphere at a ramp of 2 °C/min and was held at that temperature for 2 h. After being cooled to room temperature, the obtained sample was defined as Ru_1Co_{20}/HAP catalyst and then transferred to the reaction mixture without being exposed to air.

Reaction tests. In the typical reaction for reductive amination of furfural, 0.5 mmol furfural (FAL, purified by bulb-to-bulb distillation under reduced pressure), 50 mg catalyst (without being exposed to air), and 5 g *p*-xylene (Alfa, anhydrous) were put into polytetrafluorethylene **c**hamber in an autoclave (Parr reactor with a volume of 50 mL). After sealing the autoclave, the autoclave was purged with NH_3 for three times, and charged with 0.5 MPa NH_3 and 1 MPa H_2 at room temperature. Then the reaction mixture was stirred at a rate of 800 r/min and heated at 100 °C for 6 h subsequently heated for 20min to 180 °C for 14 h. After the reaction, the liquid-phase products were analyzed with a GC system (Agilent 7890A) equipped with a HP-5 column (30 m × 0.25 um × 0.25 mm i.d) and a FID detector by using dodecane as an internal standard.

The conversion of FAL (X_{FAL}) and the yield of piperidine ($Y_{\mathbf{piperidine}}$) were calculated using the following equations:

$\mathbf{X_{FAL}}$ **(%)** = $(mol_{FAL\ consumed})/\ (mol_{FAL\ fed}) * 100$

$\mathbf{Y_{piperidine}}$ **(%)** = $(mol_{piperidine\ produced})/\ (mol_{FAL\ fed}) * 100$

Piperidine production rate in table 1 ($mol_{2a}·mol_{Ru}^{-1}·h^{-1}$) = produced piperidine (mol) / (50 mg × 10^{-3} × Ru loading (wt%) / (100 * 101) × 20 h)

The production process of pyridine: After the typical reaction, the reaction mixture was stirred under room temperature for 30 mins to release the NH_3. Then, the reaction solvent was stirred under room temperature to remove dissolved NH_3. Then the autoclave was further purged with N_2 for three times and charged with 2 MPa N_2 at room temperature. Then the reaction mixture was stirred at a rate of 800 r/min and heated at 240 °C for 24 h. After the reaction, the liquid-phase products were analyzed with a GC system (Agilent 7890A) equipped with a HP-5 column (30 m × 0.25 um × 0.25 mm i.d) and a FID detector by using dodecane as an internal standard.

The production process of piperidine hydrochloride: After the typical reaction, the Ru_1Co_{20}/HAP catalyst was removed by centrifugation from the reaction mixture. Then, 5 ml 2M aqueous hydrochloric acid solution was added to the solution, and the piperidine hydrochloride was extracted in H_2O phase. The pure piperidine hydrochloride solid was obtained by evaporation.

The production process of Artane: 0.5 mmol obtained piperidine hydrochloride, 0.5 mmol acetophenone, 1 mmol paraformaldehyde were added into 50 mL 0.1 M HCl ethanol solution and refluxed at 100 °C for 6 h. The mixture was evaporated and the obtained solid was washed by 50 mL diethyl ether, and then collected by filtration and alkalized

with 1M NaOH, finally to obtain the β-piperidinopropiophenone. Then, the β-piperidinopropiophenone and 2 mL dry THF were added in Schlenk tube under anhydrous and oxygen-free conditions in ice bath, the 1mL 2M cyclohexylmagnesium chloride in ether was added drop by drop to the solution, to get the final product Artane.

The actual Co and Ru loadings were determined by inductively coupled plasma spectroscopy (**ICP-OES**) on an IRIS Intrepid II XSP instrument (Thermo Electron Corporation).

H_2-TPR was carried out with a Micromeritics AutoChem II 2920 System. 0.10 g of the calcined sample was loaded in a quartz reactor, heated in Ar flow at 300 °C for 1 h with a ramp of 10 °C/min, and then cooled down to 50 °C. The reactor was flushed with 10% H_2/Ar to reach a stable background. Then the sample was heated to 800 °C at a rate of 10 °C/min in 10 vol% H_2/Ar with a flow rate of 30 mL/min.

The **high-angle annual dark-filed scanning transmission electron microscopy (HAADF-STEM)** and **energy dispersive X-ray spectroscopy (EDS)** experiments were performed on a JEOL JEM-2100F microscope operated at 200 kV, equipped with an Oxford Instruments ISIS/INCA energy-dispersive X-ray spectroscopy (EDS) system with an Oxford Pentafet Ultrathin Window (UTW) Detector. Before microscopy examination, the sample was ultrasonically dispersed in ethanol for 15-20 min, and then a drop of the suspension was dropped on a copper TEM grid coated with a thin holey carbon film.

In-situ X-ray diffraction (XRD) analysis was carried out on a PANalytical X'pert diffractometer using Cu Kα radiation source (λ = 0.15432 nm) with a scanning angle (2θ) of 10°- 80°, operated at 40 kV and 40 mA. The Ru_1Co_{20}/HAP catalyst was transferred to the chamber with H_2 flow of 20 mL/min, and the XRD spectra were collected with elevating the temperature at every stage of 50 °C.

In-situ X-ray photoelectron spectroscopy (XPS) spectra were obtained on a Thermo ESCALAB 250 X-ray photoelectron spectrometer equipped with Al Kα excitation source and with C as internal standard (C 1s = 284.6 eV). The Ru_1Co_{20}/HAP catalyst was transferred to the XPS chamber with 10vol% H_2/Ar flow of 10 mL/min for 1 h, and the XPS spectra were collected after being cooled to room temperature.

Quasi-in-situ X-ray absorption spectra (XAS) including X-ray absorption near edge structure (XANES) and extended X-ray absorption fine structure (EXAFS) at Ru K-edge of the samples were measured at the beamline 14W of Shanghai Synchrotron Radiation Facility (SSRF) in China. The output beam was selected by Si (311) monochromator, and the energy was calibrated by Ru foil. The data were collected at room temperature under transmission mode. Athena software package was employed to process the XAS data. The samples were reduced at 400 °C for 2 h and directly sealed in the Kapton film without being exposed to air.

NMR spectra were recorded at room temperature in $CDCl_3$ on 300/400 MHz Bruker DRX-400 NMR spectrometers.

Computational Methods. All DFT calculations were carried out with the Vienna Ab-initio Simulation Package (VASP).[1] The electron exchange and correlation energy was treated by generalized gradient approximation (GGA) based on the Perdew−Burke−Ernzerhof (PBE) functional.[2] Projector-augmented-wave (PAW) potential was employed to describe the interaction between ions and electronss.[3] The Kohn–Sham wave functions was expanded with a plane-wave basis set with a cut-off energy of 400 eV.[4] The thermodynamically Ru_1/Co (001) surface were used to simulate the Co surface of Ru_1Co_{20}/HAP catalyst.[5] The Ru_1/Co(001) were modeled with four-layer-thick slabs. The

upper two layers of the slabs together with the adsorbates were allowed to relax, whereas the bottom two layers fixed at bulk position during the structure optimizations. A 15 Å of vacuum layer was used to separate the surface from the periodic image. The Brillouin zone was sampled by (3×3×1) Monkhorst−Pack k-point mesh.[6] The convergence criterion for the electronic self-consistent iteration and force were set to10^{-5} eV and 0.05 eV/Å, respectively. The adsorption energies (E_{ads}), were calculated by $E_{ads} = E_{adsorbate+surface} - (E_{adsorbate} + E_{surface})$, where $E_{adsorbate+surface}$, $E_{surface}$ and $E_{adsorbate}$ are total energy of surface covered with adsorbates, the energy of clean surface and the energy of free adsorbate, respectively.

Results and Discussion

Supplementary Figure 1

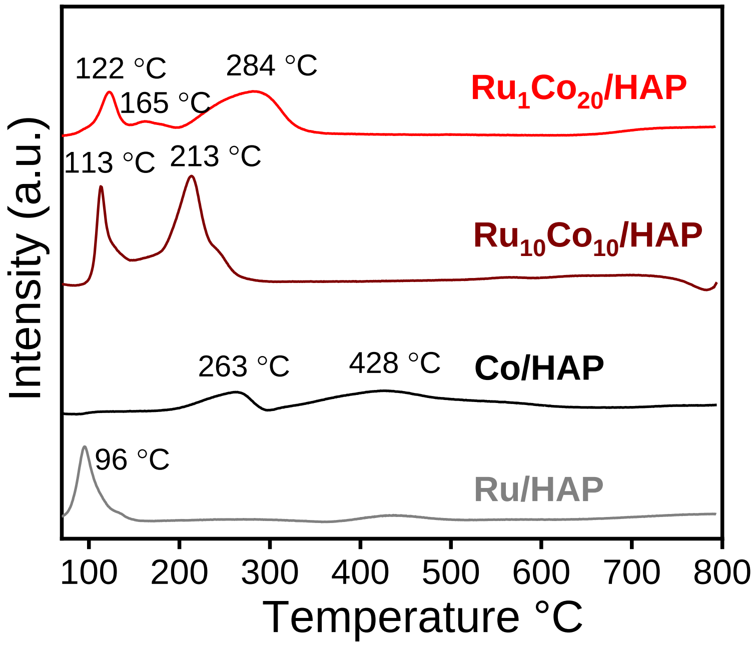

Supplementary Figure 1. H_2-TPR profiles of Ru_xCo_y/HAP samples. The H_2-temperature-programmed reduction (H_2-TPR) profile of the calcined Ru_1Co_{20}/HAP presents two major peaks at 122 °C and 284 °C, which are attributed to the reduction of RuO_2 and Co_3O_4, respectively. In addition, a minor peak occurs at 165 °C which is not observed for either monometallic Ru/HAP and Co/HAP samples or bimetallic $Ru_{10}Co_{10}$/HAP.

Supplementary Figure 2

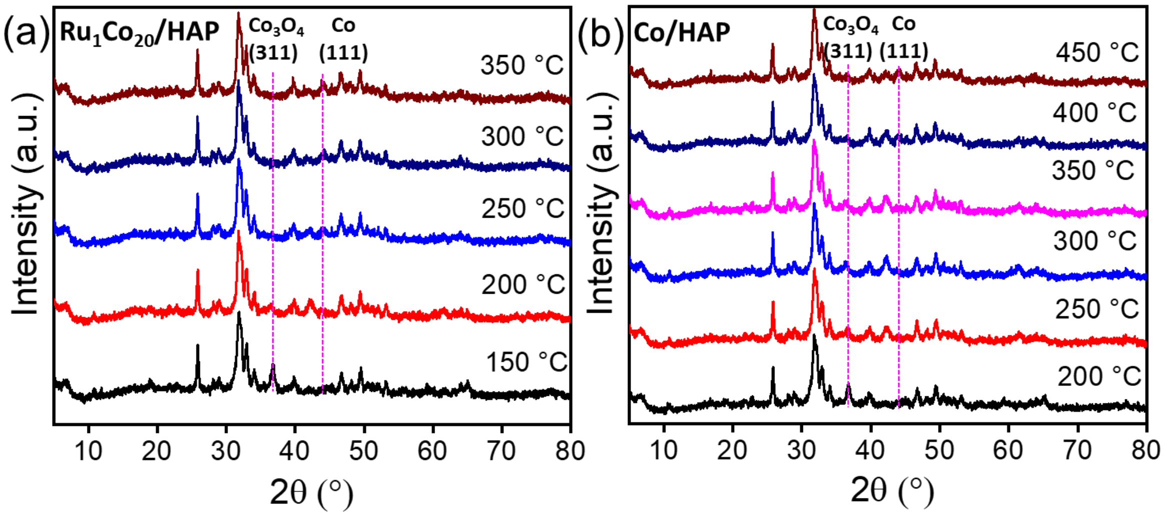

Supplementary Figure 2. *In-situ* XRD profiles of (a) Ru_1Co_{20}/HAP and (b) Co/HAP samples. The *in-situ* X-ray diffraction patterns of the Ru_1Co_{20}/HAP under H_2 atmosphere show the appearance of a metallic Co peak and concurrent disappearance of the Co_3O_4 peak when the reduction temperature reaches 250 °C, which is 200 °C lower than Co/HAP.

Supplementary Figure 3

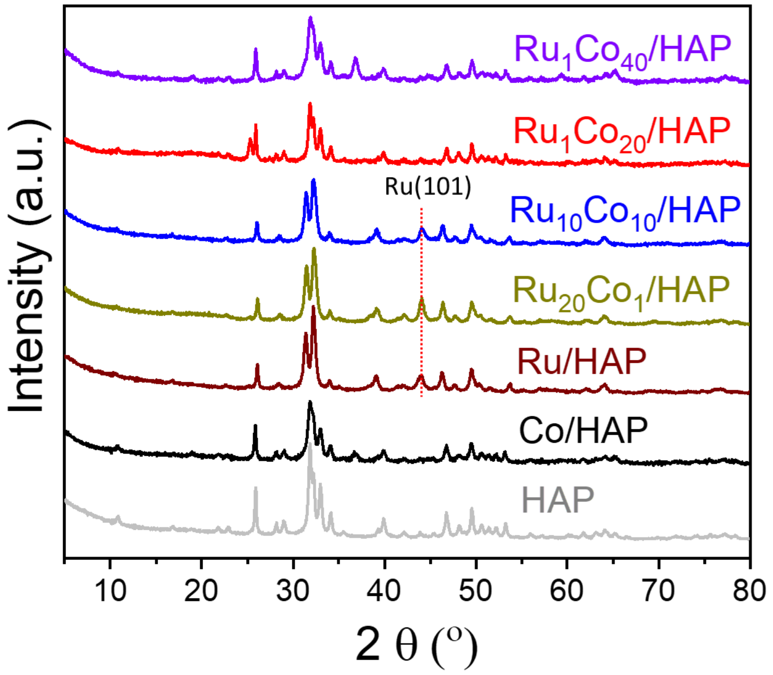

Supplementary Figure 3. *Ex-situ* XRD of Ru_xCo_y/HAP catalysts. The absence of any reflections of Ru in the XRD pattern of reduced Ru_1Co_{20}/HAP suggests the high dispersion of Ru species. For comparison, the reflection of metallic Ru is evident in $Ru_{10}Co_{10}$/HAP, indicating the formation of large Ru NPs.

Supplementary Figure 4

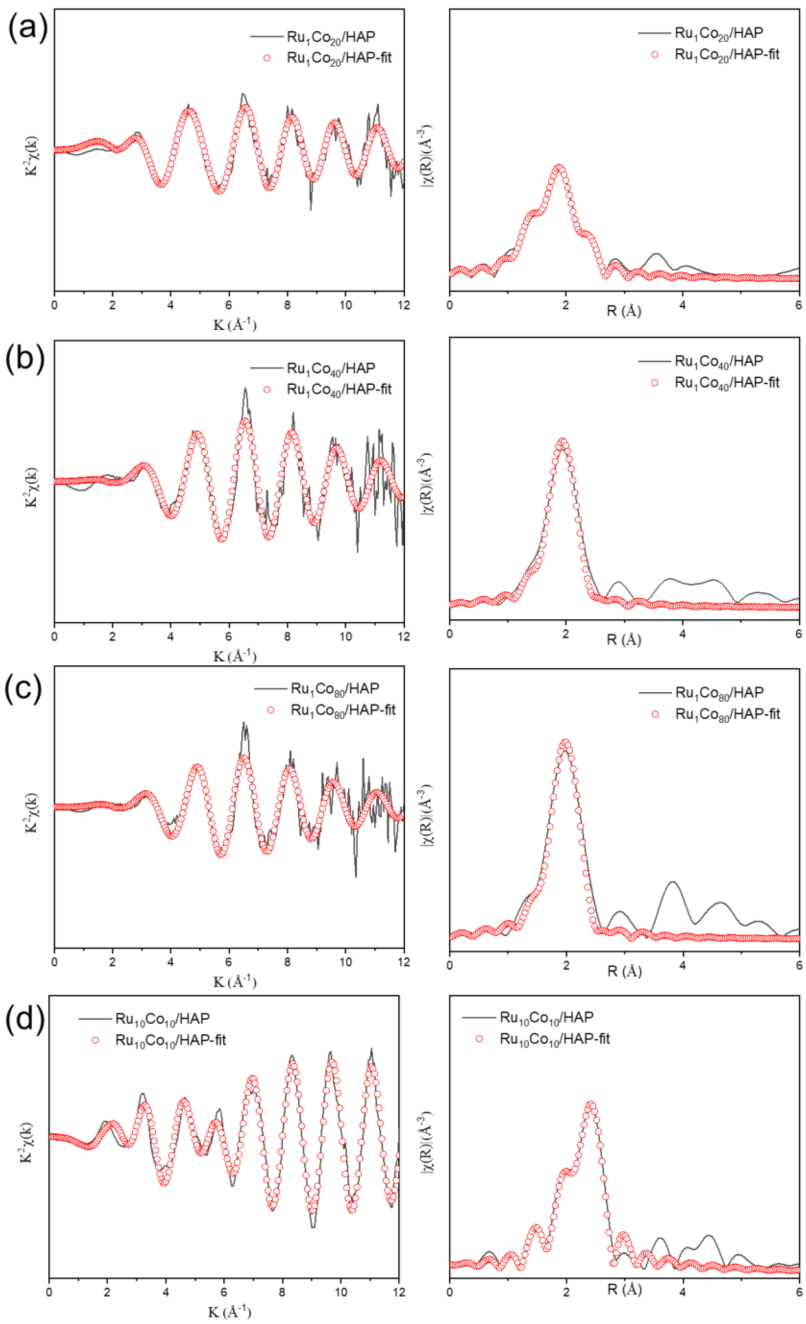

Supplementary Figure 4. The corresponding FT-EXAFS fitting curves of (a) Ru_1Co_{20}/HAP, (b) Ru_1Co_{40}/HAP, (c) Ru_1Co_{80}/HAP, and (d) $Ru_{10}Co_{10}$/HAP catalysts. The best-fitted EXAFS result of the Ru_1Co_{20}/HAP sample reveals Ru-Co shell at 2.52 Å with coordination number (CN) of 6.9 and Ru-Ru shell at 2.59 Å with CN of 3.4; the fitted results of the Ru_1Co_{40}/HAP and Ru_1Co_{80}/HAP samples reveal Ru-Co shell at 2.46 Å with CN of 5.3 and Ru-Co shell at 2.48 Å with CN of 5.7, respectively; and the best-fitted result of the $Ru_{10}Co_{10}$/HAP presents Ru-Ru coordination at 2.66 Å with CN of 8.1 and Ru-Co coordination at 2.54 Å with CN of 1.1.

Supplementary Figure 5

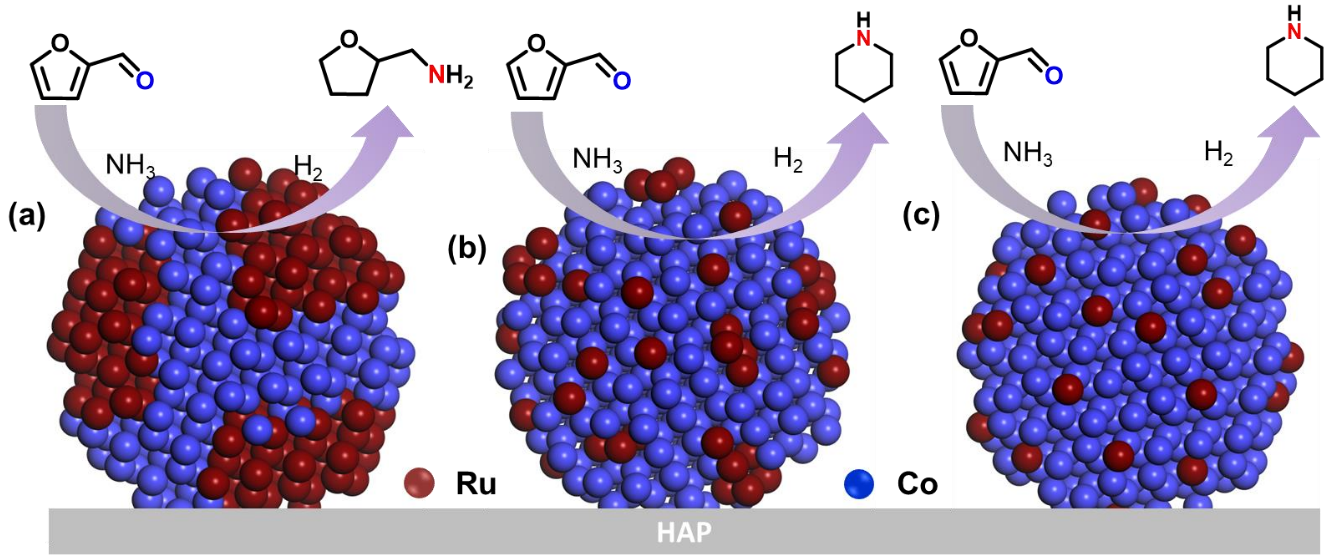

Supplementary Figure 5. The postulated structures represent $Ru_{10}Co_{10}$/HAP (a), Ru_1Co_{20}/HAP (b), and Ru_1Co_y/HAP (y = 40, 80, and 160) (c) catalysts, respectively**.** It can be reasonably postulated that Ru_1Co_{20}/HAP contains Ru single atoms and small Ru clusters, and $Ru_{10}Co_{10}$/HAP contains Ru nanoparticles. While the Ru atoms in the Ru_1Co_y/HAP catalysts (y = 40, 80, and 160) are sufficiently isolated by the neighbouring Co atoms.

Supplementary Figure 6

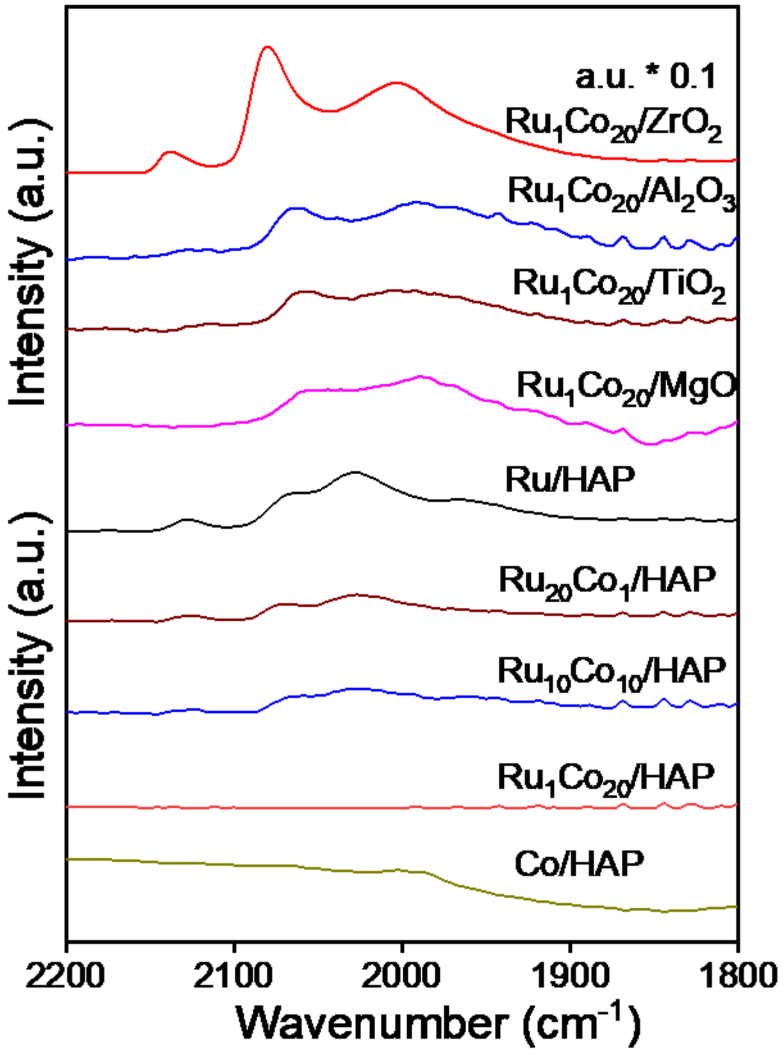

Supplementary Figure 6. CO-probed FT-IR spectra for Ru_xCo_y samples. Both Ru/HAP and $Ru_{10}Co_{10}$/HAP show bands at around 2139~2100, 2081~2050, and 2003~1850 cm^{-1}, which could be assigned to the stretching vibrations of Ru^{n+}-$(CO)_x$, Ru^0 NP-CO, and Ru_2^0-CO[43], respectively, indicative of the predominance of the Ru surface. In contrast, no bands are detected for the Ru_1Co_{20}/HAP sample, which is probably due to the formation of the surface single-atom alloy Ru_1Co_{NP} structure that suppresses CO adsorption. More interestingly, when comparing different supported Ru_1Co_{20} catalysts, it is found that all the samples exhibit similar CO absorption bands compared to the pure Ru/HAP except for the HAP supported one, which also suggests that the HAP support plays a unique role in promoting the formation of a surface single-atom alloy Ru_1Co_{NP} structure.

Supplementary Figure 7

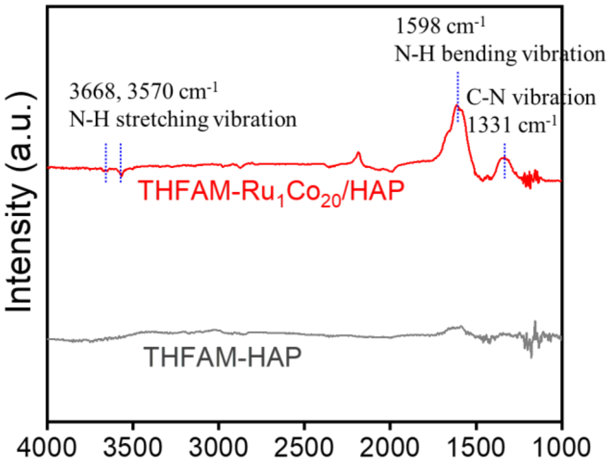

Supplementary Figure 7. THFAM-adsorbed IR spectroscopy on Ru_1Co_{20}/HAP and HAP under 180 °C. IR test process: The Ru_1Co_{20}/HAP or HAP materials were filled in *in-situ* IR cell and reduced by hydrogen under 400 °C for 2h, then the gas was switched to argon and temperature was increased to 410 °C for 30 min to remove adsorbed H species on materials. Further, the temperature was decreased to 180 °C and kept stable. Finally, the THFAM was bubbled with Ar to IR cell and IR signal was monitored after subtracting the background of materials.

Supplementary Figure 8

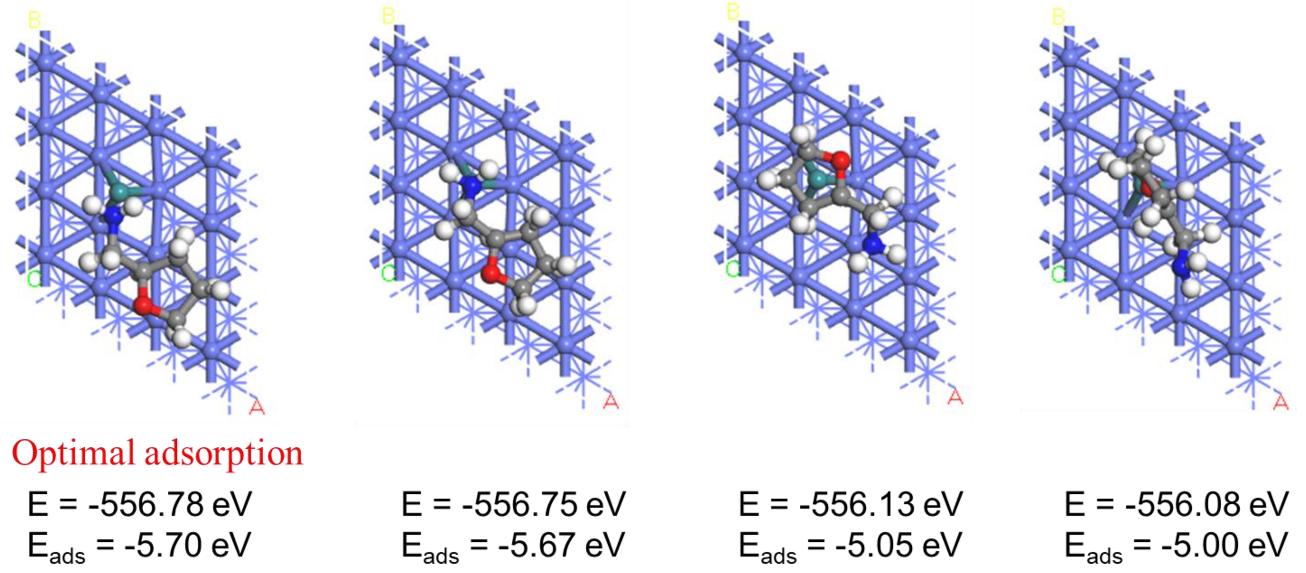

Supplementary Figure 8. Optimized THFAM adsorption configurations on Ru_1/Co surface. The optimized THFAM adsorption configurations show that THFAM is strongly adsorbed on the Ru_1/Co (001) surface with the N atom of the amino group binding to the Ru_1 atom while the tetrahydrofuran ring binds to the Co surface, and the adsorption energy is -5.70 eV.

Supplementary Figure 9

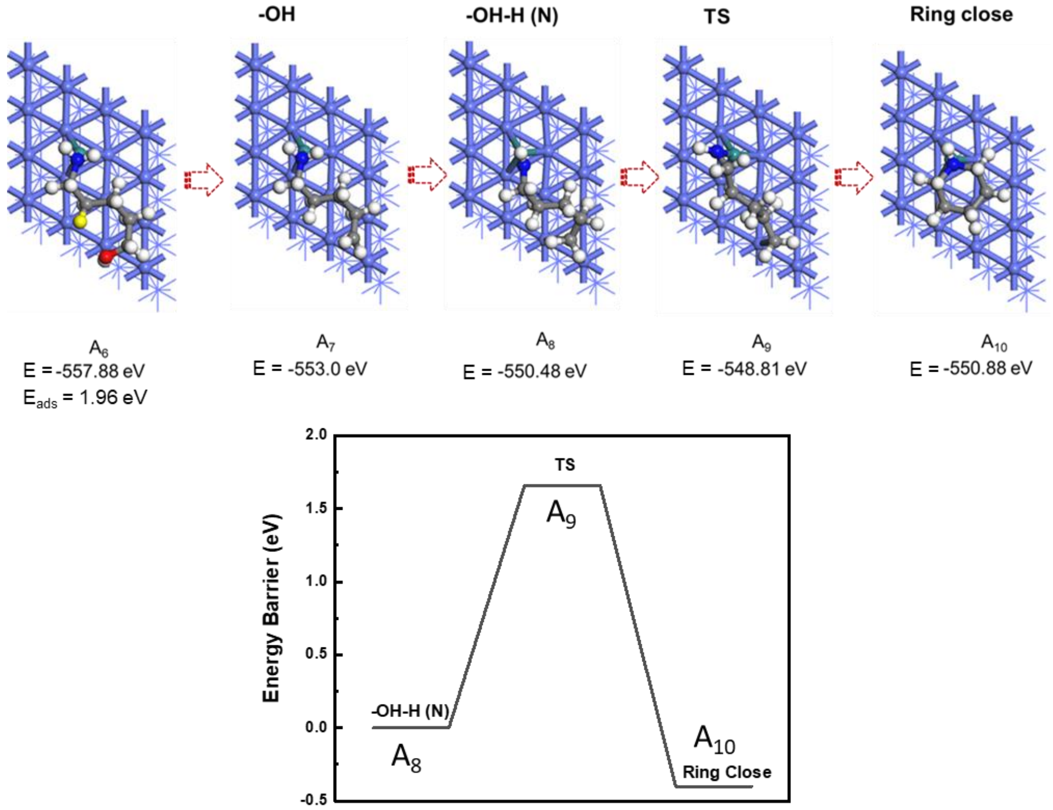

Supplementary Figure 9. Detailed adsorption configuration and energy for ring-closing process on Ru_1/Co. The energy barrier (1.66 eV) of 5-amino-1-pentanol to TS (A_9) is much lower than desorption barrier (1.96 eV) of 5-amino-1-pentanol, which indicated that the adsorbed 5-amino-1-pentanol would be directly ring-closed to final product piperidine once it was produced rather than desorbed, which also explains the reason of its absence in kinetic profile.

Supplementary Figure 10

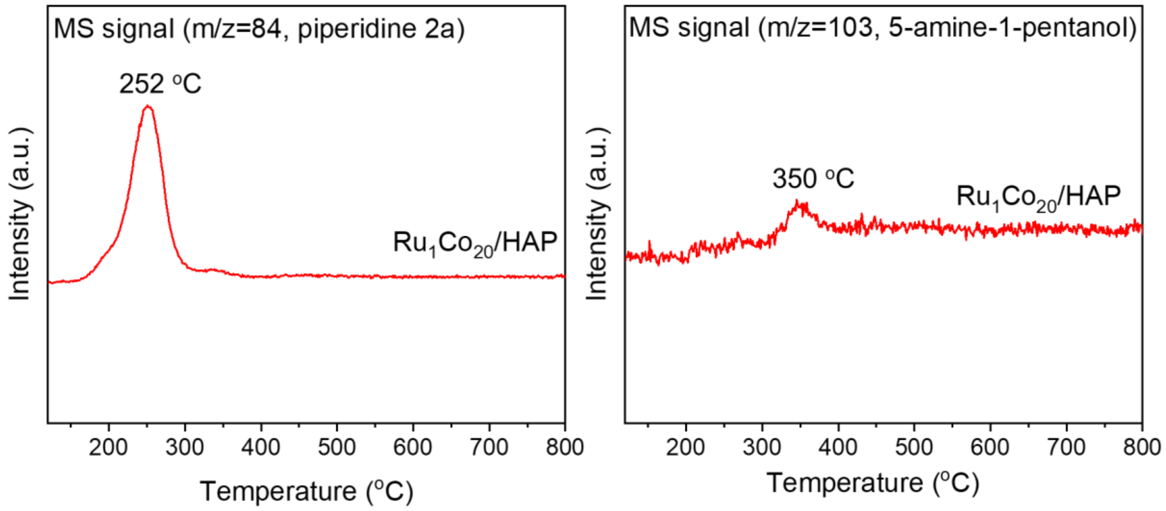

Supplementary Figure 10. The temperature-programmed desorption of 5-amino-1-pentanol on Ru_1Co_{20}/HAP catalyst. (The 5-amine-1-pentanol was adsorbed at 120 °C, and then desorbed under N_2 atmosphere with temperature increasing rate of 10 °C/min). Temperature-programmed desorption (TPD) of 5-amino-1-pentanol was performed under N_2 in the presence of the Ru_1Co_{20}/HAP catalyst. In this experiment, piperidine is detected at 252 °C, while 5-amino-1-pentanol is desorbed at 350 °C. Obviously, the ring-closing of 5-amino-1-pentanol *via* dehydration proceeded before it was desorbed from the catalyst surface.

Supplementary Figure 11

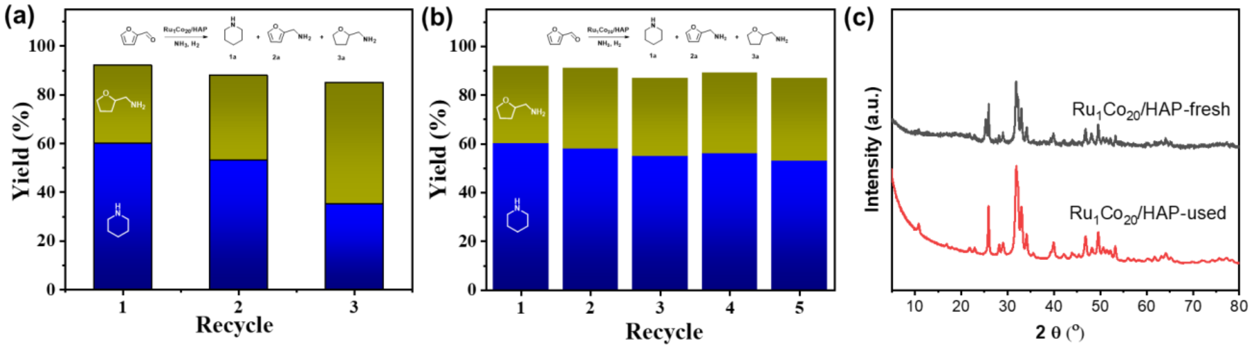

Supplementary Figure 11. Catalyst reusability (a) without and (b) with H_2 reduction[a] for used Ru_1Co_{20}/HAP catalyst, (c) XRD patterns of fresh and used Ru_1Co_{20}/HAP catalyst. Reaction condition: 50 mg Ru_1Co_{20}/HAP, 0.5 mmol furfural, 5 g *p*-xylene, 0.5 MPa NH_3,1 MPa H_2, 100 °C, 6 h, subsequently, 180 °C, 4 h, dodecane as internal standard; [a]The Ru_1Co_{20}/HAP was reduced at 400 °C before next run.

Supplementary Figure 12

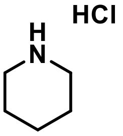

^{1}H NMR (400 MHz, DMSO) δ 9.20, 4.30, 2.97, 2.96, 2.94, 2.94, 2.92, 1.70, 1.69, 1.67, 1.66, 1.65, 1.56, 1.55, 1.53, 1.52, 1.50.
^{13}C NMR (101 MHz, DMSO) δ 43.85, 22.44, 22.18.

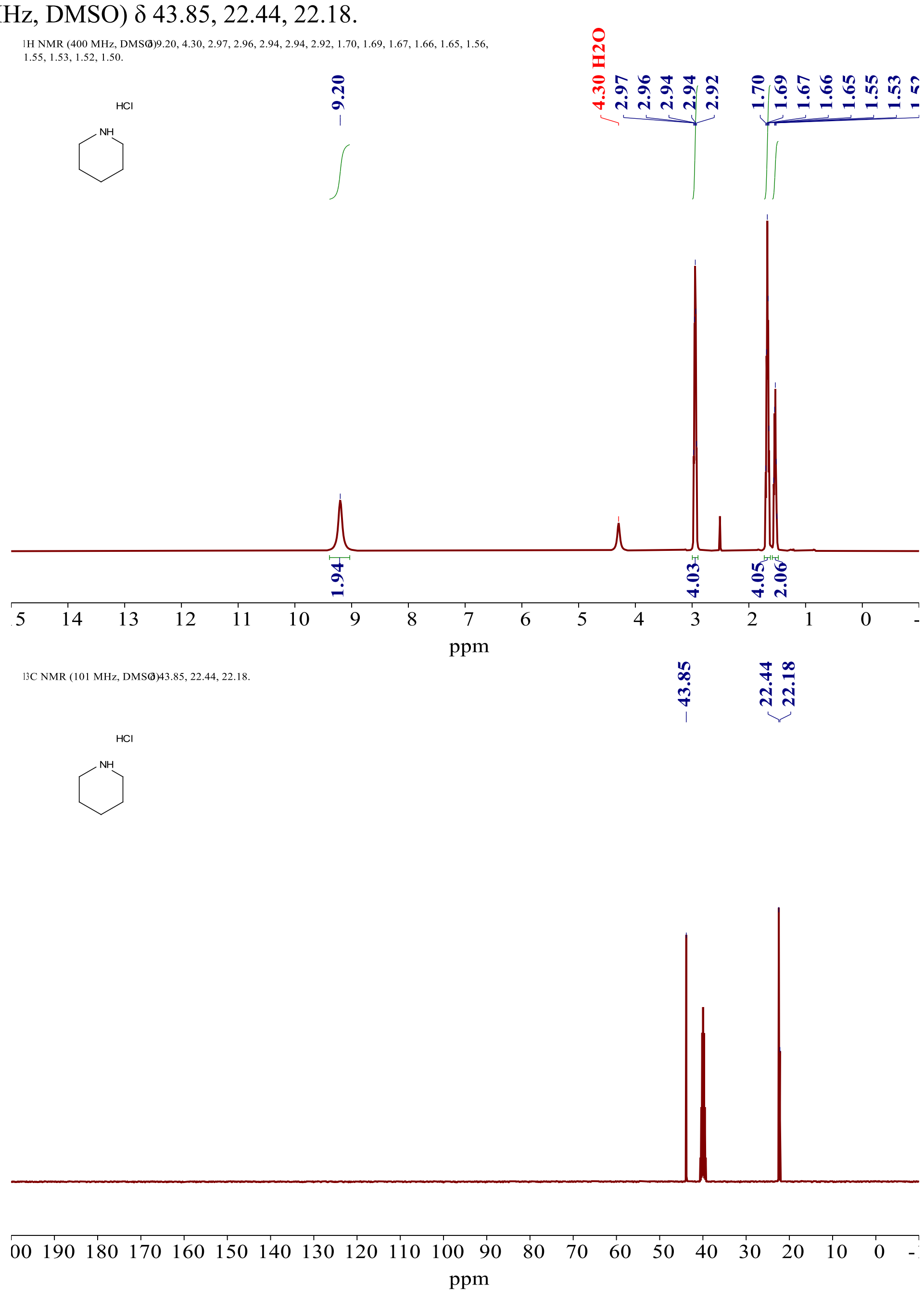

Supplementary Figure 12. ^{1}H NMR and ^{13}C NMR spectra of piperidine hydrochloride.

Supplementary Figure 13

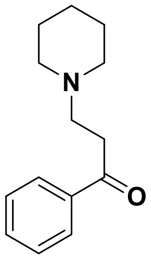

^{1}H NMR (300 MHz, $CDCl_3$) δ 7.99-7.77 (m, 2H), 7.52-7.43 (m, 1H), 7.41-7.33 (m, 2H), 3.28-3.02 (m, 2H), 2.84-2.61 (m, 2H), 2.45-2.29 (m, 4H), 1.44 (ddt, J = 47.4, 11.1, 5.5 Hz, 6H).

^{13}C NMR (75 MHz, $CDCl_3$) δ 199.38, 136.98, 133.03, 128.59, 128.03, 54.62, 53.92, 36.38, 25.98, 24.27.

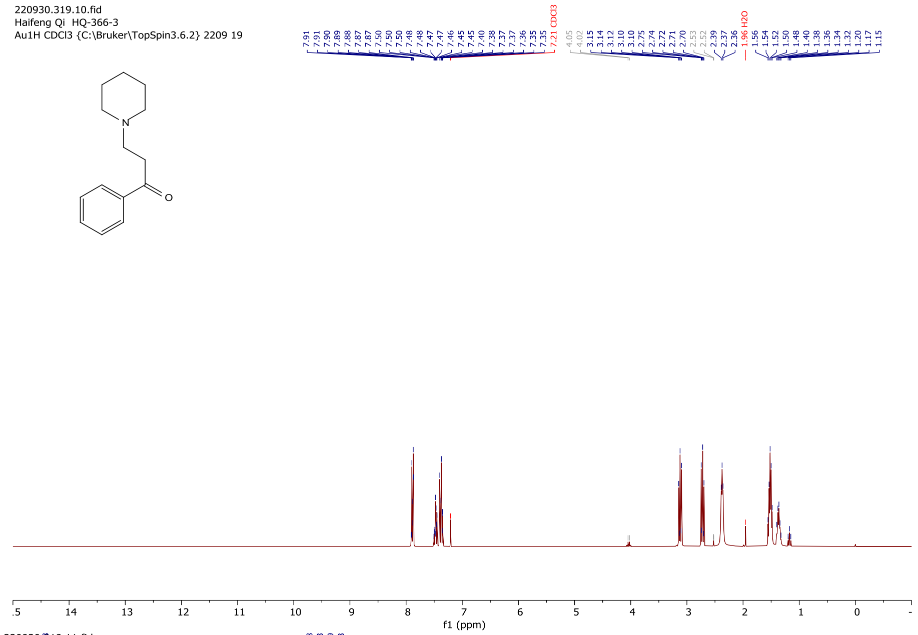

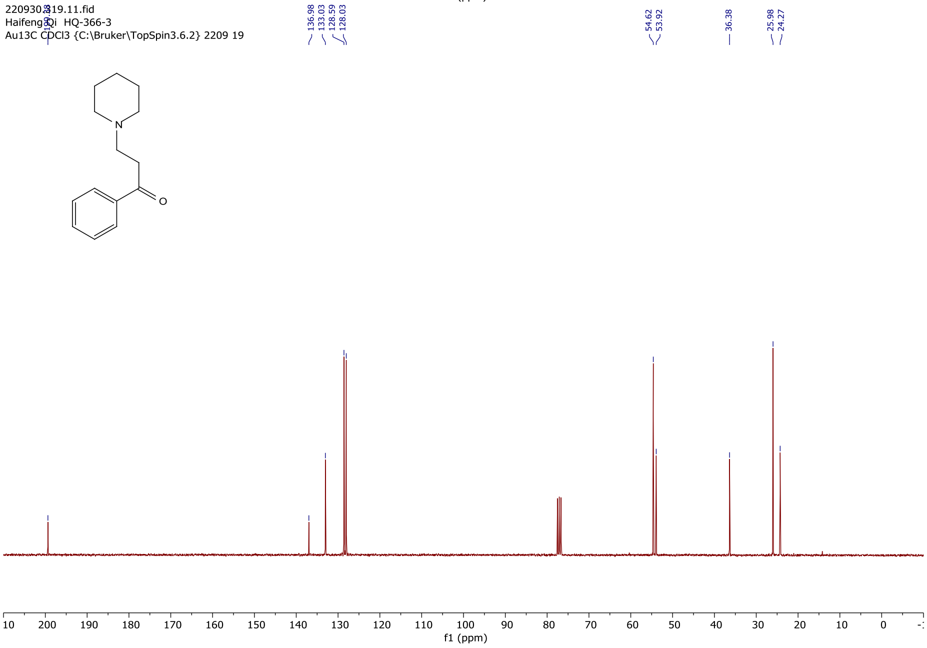

Supplementary Figure 13. ^{1}H NMR and ^{13}C NMR spectra of β-piperidinopropiophenone.

Supplementary Figure 14

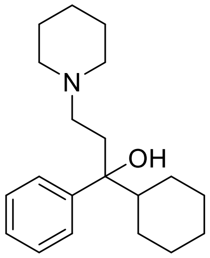

^{1}H NMR (300 MHz, $CDCl_3$) δ 7.44-7.34 (m, 2H), 7.35-7.27 (m, 2H), 7.24-7.12 (m, 1H), 2.69-2.31 (m, 2H), 2.34-2.12 (m, 3H), 2.11-1.82 (m, 3H), 1.82-1.35 (m, 11H), 1.32-0.95 (m, 6H). ^{13}C NMR (75 MHz, $CDCl_3$) δ 147.32, 127.71, 126.34, 125.81, 79.98, 55.62, 54.51, 49.16, 33.07, 27.26, 27.02, 26.89, 26.67, 26.11, 24.26. [6]

HR-MS (EI) m/z calcd for ($C_{20}H_{31}NO^{+}$) [M^{+}], expected mass: 302.2484, observed mass: 302.2485.

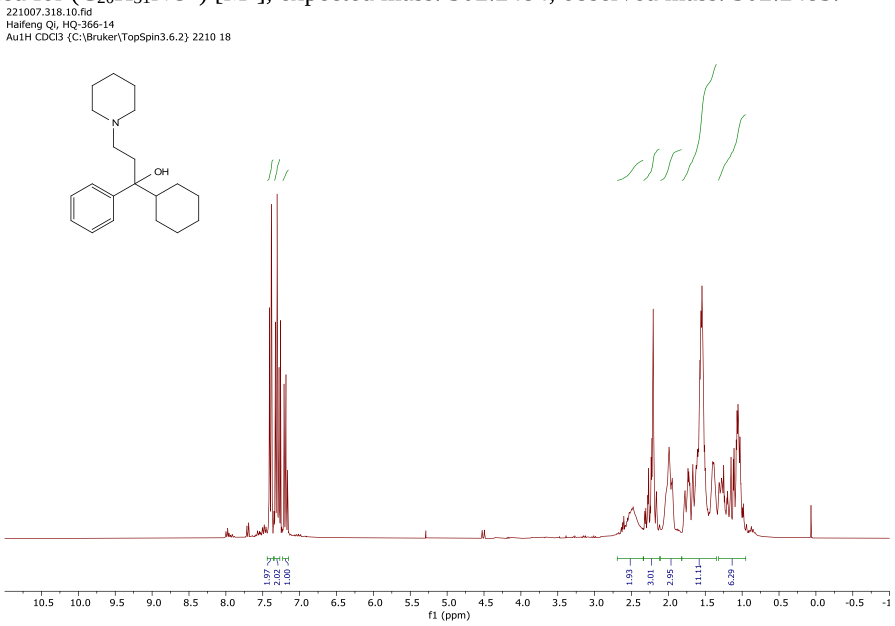

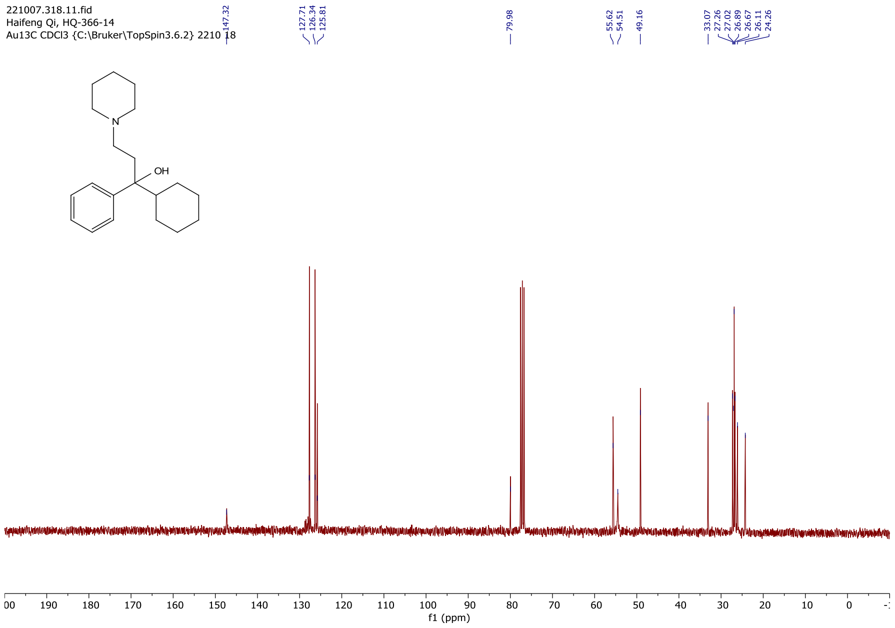

ESI-TOF Accurate Mass Report
File:22102402
Vial:1:F,2
Description:MeOH/0.1%HCOOH in H2O 90:10

Sample Name:HQ-366-v
Date:24-Oct-2022

UserName:Haifeng Qi
Time:10:29:36

Page 2

Sample Report:

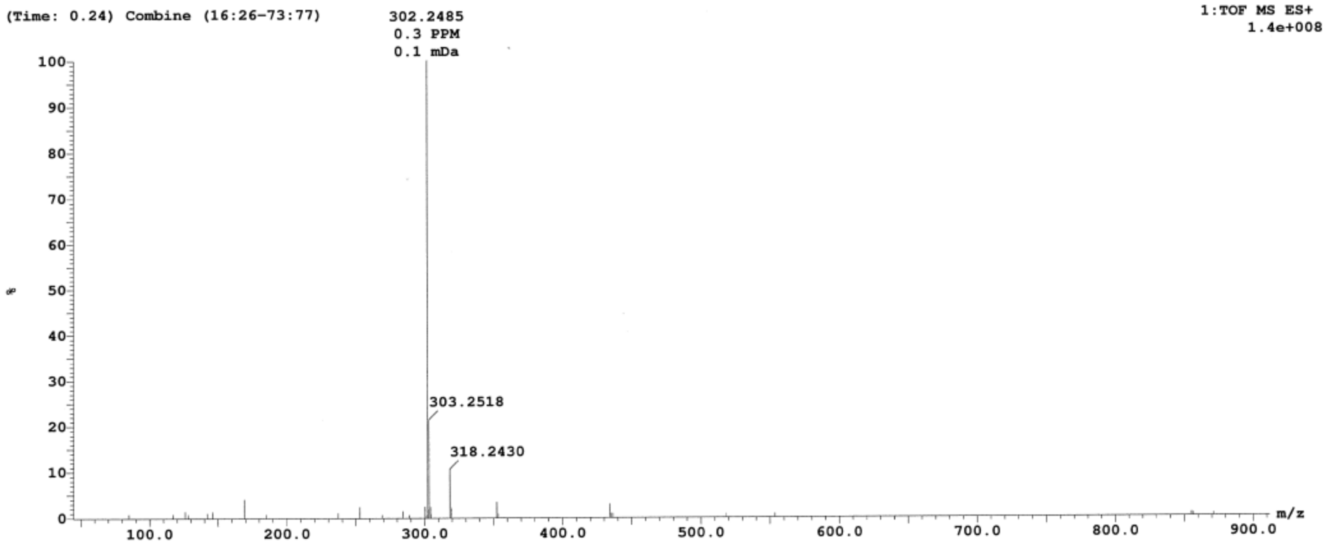

Supplementary Figure 14. ^{1}H NMR, ^{13}C NMR and HR-MS spectra of Artane. [7]

Supplementary Table 1. Catalytic performance of Ru_1Co_{20} on different supports under 220 °C.[a]

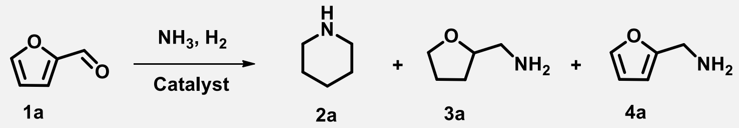

Entry	Catalysts	T. (°C)	Yield (%)[b]		
			2a	3a	4a
1	$Ru_{10}Co_{10}$/HAP	220	n.d.	92	n.d.
2	Ru_1Co_{20}/Al_2O_3	220	n.d.	56	n.d.
3	Ru_1Co_{20}/MgO	220	n.d.	59	n.d.
4	Ru_1Co_{20}/TiO_2	220	n.d.	83	n.d.
5	Ru_1Co_{20}/ZrO_2	220	n.d.	88	n.d.

[a] Reaction conditions: 50 mg catalyst, 0.5 mmol furfural, 5 g *p*-xylene, 0.5 MPa NH_3, 1 MPa H_2, 100 °C for 6 h and 220 °C for 14 h, dodecane as internal standard; the subscripts of Co and Ru represent the Co/Ru molar ratio. [b] the conversions of furfural were all >99%, and other products were oligomers and polymers.

Supplementary Table 2. Hydrogenation of tetrahydrofurfurylamine over supported Ru_xCo_y catalysts.

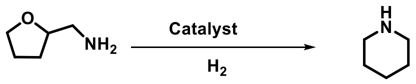

3a 2a

Entry	Catalysts	Yield (%)
		2a
1	Ru_1Co_{20}/HAP	96
2	Ru_1Co_{20}/MgO	n.d.
3	Ru_1Co_{20}/Al_2O_3	n.d.
4	Ru_1Co_{20}/ZrO_2	n.d.
5	Ru_1Co_{20}/TiO_2	n.d.
6	Ru_1Co_{40}/HAP	97
7	Ru_1Co_{10}/HAP	91
8	Ru_5Co_{10}/HAP	n.d.
9	$Ru_{10}Co_{10}$/HAP	n.d.
10	$Ru_{20}Co_1$/HAP	n.d.
11	Co/HAP	n.d.
12	Ru/HAP	n.d.
13	HAP	n.d.
14[b]	Ru_1Co_{20}/MgO	n.d.
15[b]	Ru_1Co_{20}/Al_2O_3	n.d.
16[b]	Ru_1Co_{20}/ZrO_2	n.d.
17[b]	Ru_1Co_{20}/TiO_2	n.d.

[a]Reaction condition: 0.5 mmol tetrahydrofurfurylamine, 50 mg catalyst, 5 g *p*-xylene, 1 MPa H_2, 180 °C, 14 h, dodecane was used as an internal standard; [b]220 °C; n.d.: not detected.

Supplementary Table 3. The best-fitted EXAFS results of Ru samples[a].

Sample	Shell	CN	R (Å)	**σ^2 (10^{-2} Å^2)**	**ΔE_0 (eV)**	**r-factor (%)**
Ru foil	Ru-Ru	12	2.67	0.3	0.6	0.8
Ru_1Co_{20}/HAP	Ru-O	0.8	1.96	0.7	-15.1	0.07
	Ru-Co	4.8 (6.9*)	2.52	0.7	-15.1	
	Ru-Ru	3.4	2.59	0.7	-15.1	
Ru_1Co_{40}/HAP	Ru-Co	5.3	2.46	0.6	-19.2	0.8
Ru_1Co_{80}/HAP	Ru-Co	5.7	2.48	0.6	-15.4	1.1
$Ru_{10}Co_{10}$/HAP	Ru-O	0.9	1.98	0.3	-0.5	0.5
	Ru-Co	1.0 (1.1*)	2.54	0.3	-0.5	
	Ru-Ru	8.1	2.66	0.3	-0.5	

[a]CN is the coordination number for the absorber-backscatterer pair, R is the average absorber-backscatterer distance, σ^2 is the Debye-Waller factor, and ΔE_0 is the inner potential correction. The accuracies of the above parameters are estimated as CN, ±20%; R, ±1%; σ^2, ± 20%; ΔE_0, ±20%. The data range used for data fitting in k-space (Δk) and R-space (ΔR) are 3.0-11.3 Å^{-1} and 1.0-2.8 Å, respectively; * Ru-Co CN was calibrated by using equation of "$CN_{Ru\text{-}Co}$ = CN/Ru^0%", the Ru^0% was obtained by linear combination fitting in Supplementary Table 3.[8]

Supplementary Table 4. The molar ratio of Ru^0 and Ru^{4+} estimated by linear combination fitting (LCF) in the Ru_1Co_{20}/HAP and $Ru_{10}Co_{10}$/HAP samples.

Entry	Catalysts	Molar ratio (%)	
		Ru^0	Ru^{4+}
1	Ru_1Co_{20}/HAP	69.8	30.2
3	$Ru_{10}Co_{10}$/HAP	90.6	9.4-

Supplementary Table 5. The Co/Ru molar ratio of Ru_1Co_{20}/HAP sample obtained by ICP, XPS and XAS results.

Entry	Methods	Catalyst	Co Loading	Ru Loading	Molar Ratio (Co:Ru)
1	ICP	Ru_1Co_{20}/HAP	5.0 wt%	0.43 wt%	20:1
2	XPS	Ru_1Co_{20}/HAP	5.5 at%	1.7 at%	3.2:1
3	XAS	Ru_1Co_{20}/HAP	-	-	2.1:1

Supplementary Table 6. Comparable reaction rate of tetrafurfurylamine and 5-amino-1-pentanol to piperidine over Ru_1Co_{20}/HAP catalyst.

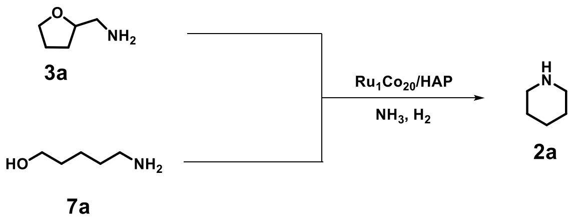

Entry	Substrate	Yield (%)
		2a
1	**3a**	19
2	**7a**	97

[a] Reaction condition: 0.5 mmol substrate, 50 mg Ru_1Co_{20}/HAP catalyst, 5 g *p*-xylene, 0.5 MPa NH_3, 1 MPa H_2, 180 °C, 1 h, dodecane was used as an internal standard.

Supplementary Table 7. Gram-scale experiment of furfural to piperidine over Ru_1Co_{20}/HAP catalyst.

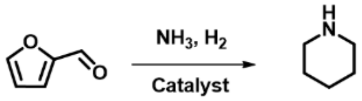

1a 2a

Entry	Substrate	Yield (%)
		2a
1	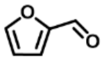 10mmol	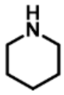 72

[a] Reaction condition: 10 mmol substrate (0.96 g), 1.0 g Ru_1Co_{20}/HAP catalyst, 2.0 g 4 Å molecular sieve (for removing produced H_2O), 25 g *p*-xylene, 1 MPa NH_3, 3 MPa H_2, 100 °C, 6 h, followed, 180 °C, 14 h, dodecane was used as an internal standard.

Supplementary Table 8. Reductive amination of 5- substituent furfural over Ru_1Co_{20}/HAP catalysts.

Entry	Substrate	Conversion (%)
		1a
1	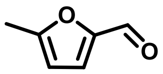	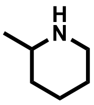 83
2	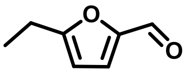	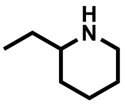 73
3	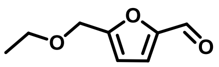	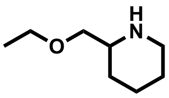 71

[a] Reaction condition: 0.5 mmol substrate, 50 mg Ru_1Co_{20}/HAP catalyst, 5 g *p*-xylene, 0.5 MPa NH_3, 1 MPa H_2, 100 °C, 6 h, followed, 220 °C, 14 h, dodecane was used as an internal standard.

Supplementary References

[1] Kresse, G., Hafner, J. Ab initio. *Phys. Rev. B* **47**, 558–561 (1993)

[2] Perdew, J. P., Burke, K., Ernzerhof, M. Generalized gradient approximation made simple. *Phys. Rev. Lett.* **77**, 3865–3868 (1996).

[3] Blöchl, P. E. Projector augmented-wave method. *Phys. Rev. B* **50**, 17953–17979 (1994).

[4] Monkhorst, HendrikJ., James D., Pack Special points for Brillouin-zone integrations. *Phys. Rev. B* **13**, 5188 (1976).

[5] Gong, X., Raval, R., Hu, P. CO dissociation and O removal on Co(0001): a density functional theory study. *Surface Science.* **562**, 247-256 (2004).

[6] Kresse, G., Furthmüller, J. Efficient iterative schemes for ab initio total-energy calculations using a plane-wave basis set. *Phys. Rev. B* **54**, 11169–11186 (1996)

[7] Paeme, G., Grimee, R., Vercruysse, A. Carbon-13 Chemical Shifts of Three Oxo-, Six Monohydroxy-, and Four Dihydroxycyclohexyl Derivatives of Trihexyphenidyl. *Bulletin Des Societes Chimiques Belges*, **92**, 995-998 (1983).

[8] M. Beale, A., M.Weckhuysen, B. EXAFS as a tool to interrogate the size and shape of mono and bimetallic catalyst nanoparticles. Phys. *Chem. Chem. Phys.* **12**, 5562-5574 (2010).
